# App Design Features Important for Diabetes Self-management as Determined by the Self-Determination Theory on Motivation: Content Analysis of Survey Responses From Adults Requiring Insulin Therapy

**DOI:** 10.2196/38592

**Published:** 2023-02-24

**Authors:** Helen N C Fu, Jean F Wyman, Cynthia J Peden-McAlpine, Claire Burke Draucker, Titus Schleyer, Terrence J Adam

**Affiliations:** 1 Center for Biomedical Informatics Regenstrief Institute Indianapolis, IN United States; 2 Richard M Fairbanks School of Public Health Indiana University Indianapolis, IN United States; 3 School of Nursing University of Minnesota Minneapolis, MN United States; 4 School of Nursing Indiana University Indianapolis, IN United States; 5 School of Medicine Indiana University Indianapolis, IN United States; 6 College of Pharmacy Department of Pharmaceutical Care & Health Systems University of Minnesota Minneapolis, MN United States; 7 Institute of Health Informatics University of Minnesota Minneapolis, MN United States

**Keywords:** diabetes app, mobile health, mHealth, diabetes, diabetic, health app, self-management, motivation, competence, autonomy, connectivity, self-determination theory, insulin, glycemic control, glucose, blood sugar, design, user need, qualitative, randomized trial

## Abstract

**Background:**

Using a diabetes app can improve glycemic control; however, the use of diabetes apps is low, possibly due to design issues that affect patient motivation.

**Objective:**

This study aimed to describes how adults with diabetes requiring insulin perceive diabetes apps based on 3 key psychological needs (competence, autonomy, and connectivity) described by the Self-Determination Theory (SDT) on motivation.

**Methods:**

This was a qualitative analysis of data collected during a crossover randomized laboratory trial (N=92) testing 2 diabetes apps. Data sources included (1) observations during app testing and (2) survey responses on desired app features. Guided by the SDT, coding categories included app functions that could address psychological needs for motivation in self-management: competence, autonomy, and connectivity.

**Results:**

Patients described design features that addressed needs for *competence, autonomy*, and *connectivity*. To promote *competence*, electronic data recording and analysis should help patients track and understand blood glucose (BG) results necessary for planning behavior changes. To promote *autonomy*, BG trend analysis should empower patients to set safe and practical personalized behavioral goals based on time and the day of the week. To promote *connectivity*, app email or messaging function could share data reports and communicate with others on self-management advice. Additional themes that emerged are the top general app designs to promote positive user experience: patient-friendly; automatic features of data upload; voice recognition to eliminate typing data; alert or reminder on self-management activities; and app interactivity of a sound, message, or emoji change in response to keeping or not keeping BG in the target range.

**Conclusions:**

The application of the SDT was useful in identifying motivational app designs that address the psychological needs of *competence*, *autonomy*, and *connectivity*. User-centered design concepts, such as being patient-friendly, differ from the SDT because patients need a positive user experience (ie, a technology need). Patients want engaging diabetes apps that go beyond data input and output. Apps should be easy to use, provide personalized analysis reports, be interactive to affirm positive behaviors, facilitate data sharing, and support patient-clinician communication.

## Introduction

### Background

Achieving treatment goals for patients with diabetes requires sustained behavioral lifestyle changes such as meal planning, monitoring carbohydrate (carb) intake and blood glucose (BG), and exercising. Diabetes apps can function as electronic care plans by helping patients plan and incorporate healthy behaviors into their daily routines [1]. The apps have been shown to lead to the improvement of glycemic control, with hemoglobin A_1c_ (a blood test measuring average BG over the past 3 months) reduction typically in the range of 0.4% to 1.9% [2-7]. The most common app functions include the documentation of BG reading, diet, and medication use; BG analysis report; data export; and email capability [8]. Visual displays of BG readings help patients link this data to their behaviors, thus facilitating behavior changes to improve glycemic control [9]. Systematic reviews have found that the effectiveness of the apps increased with greater interactivity [10,11]. Interactive feedback could be an automated message from an app algorithm [5] (eg, “you have met your BG goal setting five times this week”), a text message from a dietician who reviewed data and customized a meal plan, [3] or an alert message whenever a BG reading is out of range compared to the goal [3,4,8,12].

Despite more than 1100 apps available on the market, their adoption and use vary, possibly due to design issues [13,14] and variations in technology development [15]. To date, only a few rigorous evaluation studies of app designs have involved patients [16], and most have evaluated the quality of all available apps in the market without involving end users such as patients and clinicians [17,18]. A recent systematic review showed that patient adoption of diabetes apps weighs heavily on patient perception of benefits, ease of use, and clinician recommendation to use diabetes apps [19]. Thus, the Agency for Healthcare Research and Quality stressed the need to understand the patient perspective on the use of diabetes apps [20]. Our research question focused on adults with type 1 or 2 diabetes on insulin therapy: What diabetes app functions are helpful as explained by a theory on motivation, called the Self-Determination Theory (SDT), to promote self-management behaviors? The purpose of this study, therefore, was to describe how patients with diabetes perceive diabetes apps to address the 3 psychological needs of competence, autonomy, and connectivity as described by the SDT [21]. Our analysis also allowed us to provide evidence that would refine this theory on motivation as it applies to the use of mobile apps in the population with diabetes requiring insulin.

### Theoretical Framework

Motivation is an important factor in user experience with technology [22,23]. The SDT [21] on motivation, as expanded by Szalma [24] for motivational design on effective human-technology interaction, guided this study. The SDT posits that people are driven to engage in behaviors because they believe those behaviors will personally benefit them [25]. According to the theory, humans have 3 basic psychological needs that influence behaviors [21]. *Competence* is the need to master tasks and learn skills [26]. *Autonomy* is the need to feel in control of one’s behaviors and goals [27]. *Relatedness* or *connectivity* is the need to feel attached to other persons [26,28]. The SDT has been used in educational, business, and health care settings [29-31]. It is used to explain the human-technology interaction [24]. Ryan et al [32] reported that the ease of technology use directly and positively affected the satisfaction of psychological needs. This theory thus provides the basis for this study as we organized participant responses according to the 3 psychological needs outlined in the theory.

## Methods

### Design

This study was part of a crossover randomized laboratory trial [33] to test 2 top-rated, free commercial apps (*OnTrack* and *mySugr*), identified as the “the Best Diabetes Apps 2016” by *Healthline* [34]*.* The within-subject design helped control for patient characteristics because the same individual tested the 2 apps in random order. Quantitative measures of these diabetes apps’ usability, including user satisfaction, time, success, and accuracy rates, have been reported elsewhere [33]. The data for the analysis presented here include field notes of observations during app use, audio recordings taken during the tests, and participant responses to an electronic survey with open-ended questions that queried what app functions patients perceived as being the most useful and most important in supporting diabetes self-management.

### Ethics Approval

This study was approved by the University of Minnesota Institutional Review Board (MOD00001221).

### Participants

Using a flier posted on a bulletin board or on the web, 92 participants were recruited from the following venues: Facebook (n=46); participant referrals (n=8); Federally Qualified Health Center clinic (n=7); university campus (n=6); public housing (n=6); Craigslist (n=5); veteran’s clinic (n=4); diabetes support groups (n=3); and miscellaneous sites from a state fair, church, and library (n=7). Inclusion criteria were (1) aged ≥18 years; (2) having type 1 or type 2 diabetes; (3) having used an Android phone for 6 months or longer; (4) having used insulin therapy for 6 months or longer; (5) adequate English proficiency; and (6) smartphone proficiency (ie, they used the device for more than phone calls, emails, texting, or taking pictures). Exclusion criteria were (1) inability to read or speak English and (2) prior use of the *OnTrack* or *mySugr* app or use of any diabetes app in the past 6 months. Individuals were screened for eligibility on the phone, and written informed consent was obtained prior to the start of each study session.

### Procedures

From July to November 2017, we conducted 92 sessions of in-person tests of the apps that lasted an average of 1 hour. The testing took place in a private meeting room inside a public library or building. Participants viewed a YouTube training video posted by each app developer. They then practiced using the apps by the following protocol: (1) enter a carb intake; (2) enter an exercise activity; (3) enter an insulin dose; (4) enter a BG reading; (5) locate a BG report for specific days of the week; (6) locate a BG report for each meal; and (7) email a BG report. Then, each participant tested the 2 apps in a randomized order to carry out the same tasks listed in the practice protocol. Each participant received a US $50 gift card upon study completion.

### Data Collection

The first author (HF) kept field notes detailing her observations of participant reactions during the test of the apps and audio recorded the tests. The field notes and audio recordings were transcribed verbatim in a Microsoft Word file by a research assistant. The survey was administered on an iPad (Apple Inc.) and included questions on demographic characteristics, technology use, and diabetes history. In addition, based on the SDT [21], the survey also included questions about motivation for self-management and psychological needs for competence, autonomy, and connectivity. Details of these measures are reported in prior publication [33]. To explore participant responses to the app, the survey queried participants about their perceptions of app usability and satisfaction, preferences for a “dream” app and indications of what function(s) would be the most useful, and identification of the most important functions in a diabetes app.

### Data Analysis

Field notes, audio recordings, and survey responses were analyzed based on key constructs from the SDT [21]. The analytic team, consisting of 4 members (HF, JFW, CJP-M, and TJA), analyzed the transcripts with the aid of Dedoose [35], a web-based, qualitative data analysis software. Directed content analysis, as described by Hsieh and Shannon [36], was used. With this approach, an existing theoretical framework (SDT) was used to organize data according to predetermined categories that are aligned with key constructs in the theory: competence, connectivity, and autonomy. Data that failed to contribute to the categories were coded and used to suggest modifications or extensions of the theory. A codebook was developed based on the initial reading and updated with independent coding from an analysis team. The team reached consensus on the code definition that were clear and mutually exclusive (see Table 1 for conceptual and operation definitions for codes used).

*Competence* was conceptually defined as app features to help patients gain skills to keep BG in the target range [24]. *Competence* was operationalized as app functions to help patients understand the meaning of their data. This refers to how the app records data, analyzes data, and provides reports on which numbers are not in the target range and why. *Autonomy* was conceptually defined as app features that help patients set safe goals on diet, insulin dose, or activity level based on personal trends of BG and carb intake [24]. *Autonomy* was operationalized as app data visualization to help patients identify abnormal highs or lows, which are important for setting up reasonable targets to change behaviors associated with those abnormal readings. *Connectivity* was conceptually defined as app features to facilitate interactions between persons and the technology involved, which means enabling the sharing of home-monitored data and communicating with clinicians [24]. *Connectivity* was operationalized as app print report options, exports of data and analysis reports, and reports sent to clinicians or others through email.

Analysis occurred in several steps consistent with content analysis procedures as described by Miles et al [37]. First, based on the SDT [21], the team reviewed the conceptual definitions of the 3 main categories (eg, *competence*, *autonomy*, and *connectivity)* and, through discussion and consensus, developed operational definitions of each that were clear and mutually exclusive. See Table 1 for the conceptual and operational definitions of each of the categories. Second, a codebook was developed that outlined rules for coding data to each of the categories. The codebook was refined through several iterations of coding. Third, a table was developed that included each of the 3 categories as column headings and a column heading labeled “other” for codes that did not align with any of the categories. Data from each participant were placed on a row that was identified with the participant’s ID number. Fourth, all data were read by all team members and divided into text units (eg, coherent phrases or sentences relevant to the study purpose). The text units were coded with a label that captured the essence and, based on the coding rules, placed in the appropriate cells on the table. Fifth, the analytic team met to gather similar codes from each column into subcategories through a process of discussion and consensus. The subcategories in the 3 main columns (ie, competence, autonomy, connectivity) were described.

The team used several procedures to enhance the trustworthiness of the study findings based on criteria outlined by Lincoln and Guba [38]. First, participants were carefully chosen based on comprehensive inclusion criteria that ensured they had sufficient backgrounds to fully engage with the app testing. Second, expert consensus was achieved with a 4-member research team experienced in diabetes self-management, the SDT [21], and app use, working together to reach consensus in the interpretation and grounding of the theory of the SDT. Third, transferability was enhanced with detailed descriptions of the study population and context. Fourth, auditability was ensured with a detailed audit trial maintained in the Dedoose software chronicling all analytic decisions of the study. Finally, research bias was addressed through frequent team discussions that encouraged researcher reflexivity.

**Table 1:** Codebook on definitions of app design features.

Conceptual definition and code			Operational definition
**Help gain skill to keep BG^a^ in-target-range to promote *competence***			
	Carb^b^ counting	App feature to have carb counting help, search a food database, link carb content, and planned how much carb to eat	
	Help planning	App use to plan meal or plan behavior change in diet, meds, activity, or lifestyles as well as medication and diabetes supply due for refill. - planning action - different from alert/ reminder that is reminding a behavior	
	Monitor or track BG, carb intake, physical activities, medication use, and others	App use to monitor, track, record, or log BG, BG testing frequency, carb, activity, medication use, mood, emotional status, stress, or pain The convenience of recording data on the go or app with built in glucometer function to test and record	
	Report summary	Report or records to help understand home-monitored data as a benefit for app use, including BG averages and hemoglobin A1c statistics	
	See BG out-of-range	App analysis of BG in-target-range and out-of-range	
**Set safe and practical short- and long-term goals to promote *autonomy***			
	Trends of frequent high or low BG	Data analysis to see the trends and pattern of BG including consistency of the changes (fluctuation)	
	BG or carbs trends by time	Able to see data BG or carb in relation to time of the day	
	BG or carbs trends by days or months	Able to see BG or carb in relation days of the week, or one week - a specific format to see which day of the week Able to see BG or carb with a monthly average to give a grand overview	
**Facilitate supportive interaction between persons and technology involved to promote *connectivity***			
	Share data or reports to get feedback from clinicians on home-monitored data	Enable data upload, export, or email to send data or reports to clinicians Print reports to bring to clinic visit with clinicians	
	Support from other	Sharing with app reports with family, friend, or other non-clinician involved in their diabetes care	
**General app design to promote *positive user experience***			
	Automatic	Automatic upload data which includes sync with glucose meter, insulin pump, continuous glucose monitoring, or another medical device	
	Alert or reminders	App feature to set up alarm or reminder alert for BG testing, exercise, diet change, etc.	
	Color	Color as an important design element	
	Cost	Financial expense to use the app	
	Icon, emoji, button	Design element for app screen or app functions	
	Interactivity	Interactive feedback or response such as a sound	
	Patient-friendly	Easy to use Simple and understandable terms/icons	
	Tutorial or self-help	Tutorial, help function, or resource to help users learn to use the app	
	Voice over	Respond to voice, eliminate typing or taping of icon	

^a^BG: blood glucose.

^b^Carb: carbohydrates.

## Results

### Sample Characteristics

In all, 92 persons participated in the study. Their mean age was 54 (range 19-79) years. The majority were female (54/92, 59%), White (57/92, 62%), and college educated (61/92, 66%; Table 2).

**Figure 1:**
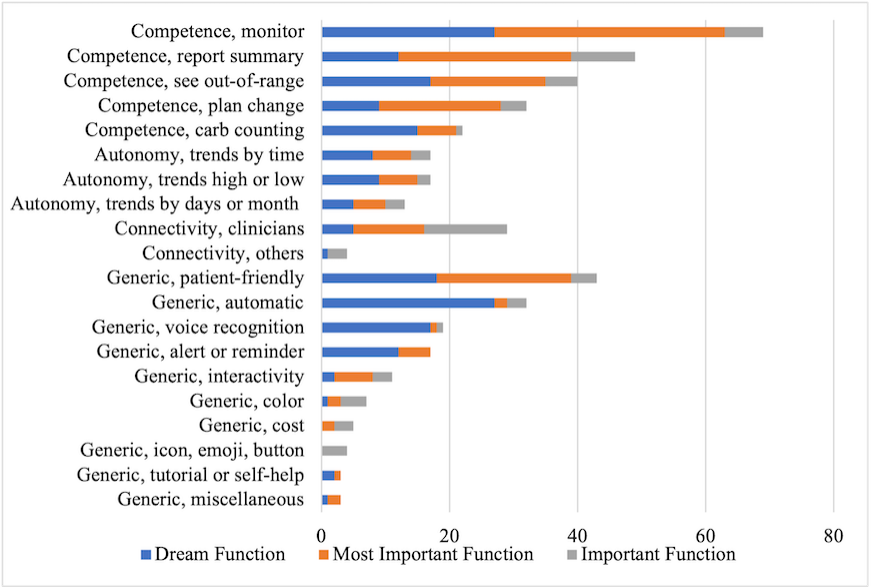
Comparison of dream function versus the most important function versus important functions in diabetes apps listed by major coding categories supportive of the Self-Determination Theory (SDT) on psychological needs (competence, autonomy, and connectivity), as well as those unsupportive of the SDT on technical needs.

Most (64/92, 70%) participants had type 2 diabetes and had used insulin for an average of 12 (SD 12) years. The participants reported a wide variety of diabetes complications including short-term memory loss; retinopathy; mobility impairment with the use of a cane, walker, or wheelchair; hemiparesis related to stroke; hand tremor; and peripheral neuropathy affecting hand dexterity. The majority (57/92, 62%) were comfortable or very comfortable using a smartphone. Additionally, 60 participants reported whether they were working (n=35) or not working (n=25)—student (n=3), retired (n=13), homeless (n=2), and disabled (n=7). Participants reported the most important app functions related to promoting competence as described by the SDT; on the other hand, what they reported as dream app functions were general app designs unrelated to the SDT (Figure 1). Of the 436 text units that were highlighted, 292 (67%) were coded to 1 of the 3 categories of needs based on the SDT [21]: competence (n=212, 48.6%), autonomy (n=47, 10.8%), and connectivity (n=33, 7.6%). The remaining 144 (33%) text units were not aligned with any of the 3 categories. The categories are discussed below.

**Table 2:** Sample characteristics (N=92).

Characteristics		Value
Age (years), mean (SD)		54 (13)
Men, n (%)		38 (41)
**Race,** **n** **(%)**		
	Alaska Native or American Indian	10 (11)
	Asian	2 (2)
	Black or African American	23 (25)
	White	57 (62)
**Highest completed education,** **n** **(%)**		
	Elementary	4 (4)
	High school or equivalent	27 (29)
	2 years of college	31 (34)
	4 years of college	19 (21)
	Graduate school	11 (12)
**Device brand,** **n** **(%)**		
	Samsung	44 (48)
	LG	19 (20)
	iPhone	8 (9)
	ZTE	7 (8)
	Motorola	6 (6)
	Other	8 (9)
**Smartphone comfort level,** **n** **(%)**		
	Very uncomfortable	23 (25)
	Neither	12 (13)
	Comfortable	33 (36)
	Very comfortable	24 (26)
**Diabetes**		
	Type 1, n (%)	28 (30)
	Type 2, n (%)	64 (70)
	Duration years, mean (SD)	17 (11)
	Insulin use years, mean (SD)	12 (12)
**Insulin use types,** **n** **(%)**		
	Insulin pump	14 (15)
	Long- and short-acting injection	46 (50)
	Long-acting injection	28 (30)
	Short-acting injection	2 (2)
	None (stopped use)	2 (2)
BG^a^ testing per day, mean (SD)		6.2 (1.4)

^a^BG: blood glucose.

### Competence

Participants found that the apps could improve their sense of competence by helping them monitor data (ranked 1st), create analysis reports (ranked 2nd), gain knowledge about reasons for out-of-range BG (ranked 4th), and plan behavior changes in self-management activities (ranked 5th), including counting carbs linked to a food library (ranked 7th; see Table 3).

Some appreciated receiving information that guided them in adjusting their insulin doses. One participant stated, “It helps me know my high and low blood sugar reading so I can adjust insulin dose. If it is real high in the morning, then at night I take more insulin. Now I do trial and error. My way is not the best.” Participants liked the automatic carb counting function. One said, “[You] take a picture [and let it] analyze for you and tell you how many carbs and everything it is.”

**Table 3:** Themes of motivational app design features as postulated by the Self-Determination Theory reported by adults with type 1 or 2 diabetes requiring insulin therapy.

Motivational design and app design features		Rank (ranged from 1-15)	Frequency (N=436), n (%)	Quotes
**Help gain skill to keep BG^a^ in-target-range to promote *competence***				
	Help record, monitor, or track BG, carb^b^ intake, physical activities, medication use, and others conveniently on a smartphone	1	69 (16)	“Ability to track sugar and foods without relying on memory” “Ability to enter as much information regarding the event (meal, exercise, etc.) as I possibly can. If I’ve exercised prior to meal or if I am sick, I want to be able to note that along with the medication or meal entry. -- tagging information to an event”
	See a report with convenient view	2	49 (11)	“Tracking my glucose readings, having at-a-glance reports and comparisons” “See blood sugar report and diet report in the apps - that way helps you maintaining your diabetes and keeping it in control”
	See out-of-range BG and explanations for abnormal readings	4	40 (9)	“The app should let you know that you are doing good or bad in any given time” “BG report when high, you can tap on it - lead you to see what you eat made it high.”
	Plan changes in diet, exercise, BG testing, and medication use	5^c^	32 (7)	“Telling me how much insulin to use with what food and exercise” “Fix your not normal readings of BG before going to see doctor”
	Carb count and provide a food library	7	22 (5)	“Adding carbs and being able to find food items with. The carbs planned out”
**Set safe and practical short- and long-term goals to promote *autonomy***				
	Trends of frequent high or low BG	9^d^	17 (4)	“Tracks your diabetes - system going up and down” “Blood glucose Trends on the home page”
	BG or carbs trends by time	9^d^	17 (4)	“Tell you when your blood sugar had a big jump” “Recording all records of bs testing, tracking foods eaten around those reading times”
	BG or carbs trends by days or months	10	13 (3)	“Ability to easily see patterns throughout the day over a period of the past 30 days” “Glucose levels compare to other hours and days. Want to know if this week, if any meal BG readings are in range.”
**Facilitate supportive interaction between persons and technology involved to promote *connectivity***				
	Quicker feedback from clinician	6	29 (7)	“Let my doctor know instead of waiting 3 months, and doctor tell me what to do to improve my diabetes” “Able to send report to doctor or print at home a paper copy to bring to an appointment”
	Support from other	14	4 (1)	“Talk with loved one [about their] data” “Within the app – meet each other weekly, get together, video, message, phone call, more secure too”

^a^BG: blood glucose.

^b^Carb: carbohydrates.

^c^Same rank as automatic feature.

^d^Same rank as set up alert or reminders.

### Autonomy

Participants found that the apps improved their sense of autonomy. They felt more self-sufficient because the apps showed if their BG was trending high or low in relation to the time (ranked 9th) and in relation to the day of the week (ranked 10th). Being provided with a data visualization of these personal patterns increased their sense of empowerment and assisted them in identifying short- and long-term goals for changing behaviors. One participant explained, “a function that easily helps me find when I most commonly have hypoglycemia.” Information provided by the apps aided their decision-making regarding how and when to change behaviors to keep BG in the target range. This could be done with data visualization; one participant stated the benefit to see “how my trends are changing.”

### Connectivity

Participants found the apps enhanced a sense of *connectivity* because the clinicians could receive emails or print reports on home-monitored data to better understand patients’ self-management behaviors (ranked 6th). One participant said, “An app that can send my numbers directly to [the doctor] if there is a concern [about frequent] lows or highs.” Participants also felt connected because of the bidirectional messaging functions of the apps. These functions supported monitoring of BG, and readings could be compared to hemoglobin A_1c_ laboratory readings in the clinic. Connectivity was also enhanced by informal coaching support from others (ranked 14th). One patient stated, “help people share what other people not understanding. (1) report, (2) sharing - support for other patients with diabetes.”

### Top General App Design

Most participants reported the necessity for a diabetes app to save time regardless of functions. They described that the app needs to be efficient and “easy,” requiring minimal user effort. They desired the app to use patient-friendly terminology and display easy-to-understand reports (ranked 3rd; see Table 4).

Automatic features (ranked 5th, same as to plan behavior change) is the integration between devices so that their data are interoperable. One participant explained, “Have this app be able to read my pump and. An app I reason I don’t use app, having an orange and apple that they don’t talk to each other. An app that easy and talk to my pump.” Voice recognition (ranked 8th) is the elimination of typing text, which was best described by one participant: “speaking function to record all data.” App alerts (ranked 9th) are helpful to remind users to do activities such as retest BG and repeat insulin for elevated BG after eating a meal. App interactivity (ranked 11th) is giving behavior confirmation as one participant explained: “You did it, completed 1 entry.” Other app designs (ranked from 12th to 15th)—color; cost; icon, emoji, or button options; tutorial or self-help; and fun, technical support, and link to pharmacy—were of interests to participants.

**Table 4:** Themes of top general app design features unsupportive of the Self-Determination Theory reported by adult with type 1 or 2 diabetes requiring insulin therapy.

App design features	Rank (ranged from 1-15)	Frequency (N=436), n (%)	Quotes
Patient-friendly	3	43 (10)	“To put language that patients could understand - small words - for example blood sugar instead of glucose.” “I like the pick and choose option but maybe more screens so there's less congestion. (Less busy screen) simple screen shot that leads to new screens. Don't like scrolling.” “Easy to read and understand the report and information you put in it - make numbers bigger”
Automatic: integration of devices plus easy view of data	5^a^	32 (7)	“Pump, and meter integration that also downloads my CGM readings to form a graph with minimal interaction from me.” “A graph to be able to connect with my meter”
Voice recognition	8	19 (4)	“Voice command to record my BG reading and carb intake” “App talks to me that my blood sugar is too high or too low”
Set up alert or reminders	9^b^	17 (4)	“Track carb, when went over the amount, it alarms you to don't eat any more carb.” “Reminder for to check your blood and make sure exercise (tell you exercise, a schedule) - like to tell you to go a walk at what time”
Interactivity	11	11 (3)	“For the app to show me the cravings for the carb, to motivate you not to eat the carb, when I eat carb, the app should go off” “Interactive apps. I really like when ‘slimy’ congratulated me or said it happens, when my sugars were not good.”
Color	12	7 (2)	“Color to differentiate functions.” “Tap in red color to give your time and more detail.”
Cost	13	5 (1)	“Don’t have to buy a meter for it.” “Willing to pay for the app if it works”
Icon, emoji, button	14	4 (1)	“More icon per se where a picture would be used instead.” “The activity (have emoji) hit emoji when you start jogging and hit emoji again to stop.”
Tutorial or self-help	15	3 (1)	“Help function - no paragraph, video to see how to use this function.” “Help function to help you use the app (like to email in the app).”
Miscellaneous: fun, link to pharmacy, technology support	15	3 (1)	Link to pharmacy order within the app and “your pharmacy deliver to you.” “For people to have a hot line, get stuck to get help technical support, a live person to help with the app. If I did not go back to last app that she showed him how to send and get gmail to send report.”

^a^Same rank as plan behavior change.

^b^Same rank as see BG (blood glucose) trends and carbs (carbohydrates) trends.

## Discussion

### Principal Findings

The aim of the research question and purpose of the study was to investigate how adults with diabetes requiring insulin therapy perceive diabetes apps based on the 3 key psychological needs described by the SDT [21]: competence, autonomy, and connectivity. Our findings provide evidence on the usefulness of the SDT in mobile health technology and describe specific app functions that address psychological needs. The results are consistent with Szalma’s [24] description of a theoretical model of motivational design based on the extension of the SDT. Newly identified categories about general app design did not fit with the SDT’s psychological needs, but they addressed the technology needs for patients to use an app with minimal effort.

### Competence

App functions help patients to record and understand data and plan behaviors as skill to keep BG in the target range. First, the convenience to track electronically whether BG is in the target range (80-130 mg/dL before eating and <180 mg/dL after eating) [39] is highly valued [40]. This is consistent with patient surveys that found diabetes apps are important for BG monitoring [41]. Understandable “Glucose Diary View” is the most practical [42]. Abnormal BG readings should be color-coded [39] and summarized into a 1-page standardized report [43]. An electronic report can increase patient knowledge to plan behavior changes such as eating right (making it easier to count carbs and plan meals) and calculating short-acting insulin dose to lower elevated BG readings due to excessive carb intake. These features are all valuable to patients because they help them to gain insight and understanding about abnormal BG readings so they can achieve competence in diabetes care, which is consistent with a study on the requirements of diabetes apps for underserved patients [44].

Carb counting is a commonly desired app function, where a smartphone takes a picture of the food; analyzes the portion size, carb content, and corresponding insulin dose; and suggests a time for insulin administration. This finding broadly supports app use to improve adherence of medical nutrition therapy [2-4,45]. Currently, many diabetes apps have low-carb diet recipes, multidevice integration, and automatic features, but the cost can be expensive. For example, *Glucose Buddy* Premium has a subscription cost ranging from US $19.99 to US $59.99 per month to access the full food database [46,47]. Future research should be undertaken to investigate ways to offset the cost of app technology such as subsidizing the expense while the health system could bill insurance for remote patient monitoring, given that the Centers for Medicare and Medicaid Services can reimburse the transmission of home-monitored data and summary report by clinic staff [48]. Offering analysis tool to count carbs and calculate insulin dose is a form of “virtual dietician.” Research is in progress to develop and test apps that leverage machine learning to perform image recognition and automate recommendations of behavior change [49].

### Autonomy

App functions of trend analysis help set safe and practical short- and long-term goals by time, day of the week, and month, which aids personalizing options to change. Participants reported the need to visualize the trends or patterns of frequent high or low BG (ie, what) by day of the week and time (ie, when). This finding is consistent with prior research showing that diabetes apps helped patients identify and incorporate healthy behaviors into their daily routine [1]. Seeing demarcations of BG changes between months, weeks, days, and time of the day is very important to show patients when dangerous BG levels occur and to set reasonable goals to change behaviors [50]. Goal or target setting helps patients plan behaviors and provides a warning when they are outside the target [51,52]. Personalizing options should include tracking mental health factors such as mood, stress, and illness, because these factors are associated with hyperglycemia and poor glycemic control. Effective self-management is important economically, since many adults diagnosed with diabetes are not able to maintain work. They exit the work force earlier (30% higher) compared to those without diabetes [53].

### Connectivity

App functions can facilitate supportive interaction by sharing data or app reports with clinicians and “loved ones” to gain support for behavior change. This is consistent with several studies that showed data sharing or showing data from the mobile devices with their clinicians during a medical visit is highly valuable for patients [50,54,55]. Greater app interactivity with a clinician appears to improve glycemic control [11,56]. A simple explanation for this finding may be that successful diabetes self-management takes teamwork [54,55]. Informal coaching support by other people or even a virtual coach in an app is valuable. Artificial intelligence could provide confirmation of positive behavior change, such as reaching a BG value in the target range, to provide immediate feedback to patients. A trial of an artificial intelligence virtual coach with 187 adults with type 2 diabetes, unfortunately, did not demonstrate a difference in changing hemoglobin A_1c_ but did improve health-related quality of life [57]. Very few long-term studies of diabetes apps have been conducted [58]. However, due to the COVID-19 pandemic, telehealth visits had an unprecedented increase in use from 0.3% in 2019 to 29.1% in 2020 among a 2019 cohort (n=1,357,029) versus a 2020 cohort (n=1,364,522) [59]. Leading companies in web-based diabetes care—*Livongo*, *One Drop*, *mySugr*, *Cecelia Health*, *Steady Health*, and *Virta Health*—noted a rise in subscribers during the pandemic [60]. Future studies using the mobile health platform for telehealth, including a diabetes app, should be undertaken.

### Top General App Functions or Features

Themes unsupportive of the SDT emerged that focused on the acceptability of general app design features. These themes did not support the SDT, but they described patients’ technology needs. The theme of being patient-friendly is highly relevant for user-centered app design. A patient-friendly app implies a match between the app and the patient’s real world [61,62], and icons and wording need to speak the users’ languages and concepts. For example, “blood sugar” is preferred to “blood glucose.” Eliminating medical jargon would decrease barriers and make it easy for patients to understand knowledge gained from using apps [50]. Automatic features to integrate devices that test BG and upload results into apps ranked in the top 5, which is consistent with a survey study among patients with type 1 diabetes, 91.6% of whom agreed that it is the most important function (n=167) [51]. Voice recognition decreases the user’s need to type data. Alert notifications can remind patients who are on multiple insulin injections and need frequent BG testing (>4 times a day). Patients desired app alerts to remind them of behavior (eg, repeat BG testing) [63]. An interactive app is about giving the patient a response to promote user interaction, not just data in and data out. A change in emoji, an app message of “good job,” or a sound are ways of interaction between the user and the technology. Color can help customize user experience. An app tutorial or technology support is an important resource to increase user confidence to interact with the app. Overall, these themes around acceptable design features are important for patient engagement to promote a positive user experience and boost patient confidence to use the technology.

### Limitations

Three major limitations in this study were (1) the laboratory setting, (2) only 2 top-rated, commercially free apps being tested, and (3) the urban population. The first weakness is that participants only used the apps once in a research visit rather than in their home setting with real data. It is possible that using the apps in the home setting would have changed participants’ opinions about the desired app features. Future work is required to establish the viability of actual app use at home and in other settings (eg, use an app for 2 weeks and attend focus groups to discuss the facilitators and barriers of app use). A second weakness is testing only 2 top-rated free apps, which may not be representative of the diabetes apps on the market. However, *mySugr* has remained in *Healthline*’s 2022 list of best diabetes apps [45], and *OnTrack* has been recommended by educators from the American Diabetes Association [46] and the University of Michigan [47]. Apps requiring payment were not included in this study. Payment for increased functionality may increase patient engagement and potentially create bias to use the app to get a return on the investment [64]. A third weakness is that the results may not be applicable to a rural population who may have no or inadequate internet service. App responsiveness may depend on the type of internet connection. Notwithstanding these limitations, this study offers valuable insight to addressing behavior needs for self-management by adults with diabetes requiring insulin therapy. Several strengths of this study include the diverse sample of racial or ethnic minority participants and a variety of diabetes complications, which increase study generalizability. Additionally, this study had a sample of 92 participants, which is much larger than most usability study sample of 30 participants.

### Conclusions

The SDT helped to explain patient perspectives on the roles of diabetes apps as an electronic tool to address their psychological needs of competence, autonomy, and connectivity in diabetes care. Our findings also validated that the 3 concepts of the SDT guided the initial coding, further analysis, and development of operational definitions. Using an app can promote competence in keeping BG in the target range through electronic monitoring of BG, creating analysis reports, and gaining knowledge about reasons for out-of-range BG to plan behavior. The app can promote autonomy to set safe and practical BG goals by showing trends of high and low readings in relation to time, day of the week, and months. An app can promote connectivity by printing reports for clinic visits or emailing reports to a clinician, thereby helping patients receive feedback from clinicians. Patient technology needs, such as being patient-friendly and requiring minimal user effort, are also important. Continued efforts are needed to understand long-term adoption of diabetes apps to support self-management by patients, as well as the integration of diabetes apps in the telehealth setting for clinicians.

## Abbreviations

BG: blood glucose
Carb: carbohydrate
SDT: Self-Determination Theory
